# A comparison between the effects of *Portulaca oleracea *seeds extract and valsartan on echocardiographic and hemodynamic parameters in rats with levothyroxine-induced thyrotoxicosis 

**Published:** 2018

**Authors:** Roghayeh Pakdel, Saeed Niazmand, Mohsen Mouhebati, Mohammad Mahdi Vahedi, Azita Aghaee, Mousa-Al-Reza Hadjzadeh

**Affiliations:** 1 *Department of Physiology, Faculty of Medicine, Mashhad University of Medical Sciences, Mashhad, Iran *; 2 *Cardiovascular Research Center, Mashhad University of Medical Sciences, Mashhad, Iran *; 3 *Cardiology Department, Ghaem Hospital, Mashhad University of Medical Sciences, Mashhad, Iran*; 4 *Department of Pharmacology, Faculty of Medicine, Mashhad University of Medical Sciences, Mashhad, Iran*; 5 *Pharmacological Research Center of Medicinal Plants, Department of Pharmacology, Faculty of Medicine, Mashhad University of Medical Sciences, Mashhad, Iran*; 6 *Division of Neurocognitive Sciences, Psychiatry and Behavioral Sciences Research Center, Mashhad University of Medical Sciences, Mashhad, Iran*

**Keywords:** Echocardiography, Hemodynamic parameters, Hyperthyroidism, Levothyroxine, Portulaca oleracea, Valsartan

## Abstract

**Objective:**

The aim of the present study was to compare the effects of *Portulaca oleracea* (Po) seeds extract and those of valsartan on cardiac function in levothyroxine (T_4_)-treated rats.

**Materials and Methods:**

Forty Wistar rats were divided into four groups (n=10): control, levothyroxine (T_4_), T_4_ plus valsartan (T_4_-Val) and T_4_ plus hydro-alcoholic extract of the *P. oleracea* seeds (T_4_-Po). Control group received normal saline. Levothyroxine (100µg/kg/day, i.p.) was administered to three other groups for 4 weeks. Valsartan (8 mg/kg/day, orally) and Po seeds extract (400 mg/kg/day, orally) were administered during the last two weeks of treatment period. At the end of the experiment, echocardiographic and hemodynamic parameters were measured and serum free T_4_, T_3_, and T_4_ were measured.

**Results:**

Administration of T_4_ for 4 weeks significantly increased serum free T_4_ levels in T_4_ group but elevations of free T_4 _levels in T_4_–Val group were not significant. Free T_4_ level decreased in T_4_–Po (p<0.01) compared to T_4_ group. Heart rate (HR), heart weight (HW), and left ventricular systolic pressure (LVSP) were significantly increased in T_4_ group compared to control group while these parameters in the other groups were not significantly different from those of control group. The reduction in HR, HW, and LVSP were more prominent in T_4_-Po group. Ejection fraction (EF) and fraction shortening (FS) were insignificantly decreased in T_4 _group compared to control group.

**Conclusion:**

These results showed that treatment of hyperthyroid rats with *P. oleracea* seeds extract was more effective than valsartan in reducing cardiac changes induced by levothyroxine.

## Introduction

Hyperthyroidism is a common endocrine disorder that produces functional and structural damages in cardiovascular system (Grais and Sowers, 2014 ▶). Cardiac manifestations of hyperthyroidism include a wide range of signs and symptoms such as tachycardia, atrial fibrillation, hypertension, widened pulse pressure, high cardiac output, increased myocardial contractility, cardiac hypertrophy and even heart failure (Danzi and Klein, 2014 ▶). Thyroid hormones (THs) directly affect cardiovascular function at the cellular level and indirectly through stimulation of autonomic nervous system. THs also decrease systemic vascular resistance but stimulate both local and peripheral renin-angiotensin systems (Jabbar et al., 2017 ▶).

Hyperthyroidism results in renin angiotensin system (RAS) activation in humans and animals (Barreto-Chaves et al., 2010 ▶). There are several reports that RAS is involved in cardiac hypertrophy due to hyperthyroidism (Hu et al., 2003 ▶; Kobori et al., 1997 ▶). A number of studies has also shown that inhibition of RAS by an angiotensin converting enzyme inhibitor or angiotensin II receptor type 1 (AT1) blocker, can prevent cardiac hypertrophy in hyperthyroidism (Asahi et al., 2001 ▶; Basset et al., 2000 ▶; Basset et al., 2001 ▶; Hu et al., 2003 ▶; Sabri et al., 1998 ▶; Wang et al., 2013 ▶). Therefore, it seems that AT1 blockers are effective in treatment of cardiac remodeling due to hyperthyroidism.

It was reported that four-week treatment with T_4_, induced experimental hyperthyroidism in Wistar rats and this was associated with cardiac hypertrophy, increases in the left ventricular end-diastolic pressure and increases in H_2_O_2_ and nitric oxide (NO) metabolites while reduction of vitamin C, GSH/GSSG ratio, and total radical trapping antioxidant potential (Araujo et al., 2008 ▶). It was reported by Araujo et al., that angiotensin receptors I and II are involved in hyperthyroidism-induced cardiac hypertrophy and treatment with antioxidant diminished expression of angiotensin II receptors and cardiac hypertrophy (Araujo et al., 2011 ▶). 

*Portulaca oleracea* (Portulacaceae family) is a fleshy annual plant found in tropical regions of the world (Yang et al., 2009 ▶). It has been widely used as an edible plant and a herbal medicine in many countries (Al-Sheddi et al., 2015 ▶). Several pharmacological properties have been reported for *P. oleracea* including neuroprotective (Wang et al., 2007 ▶), anti-proliferation (Gai Guo et al., 2016 ▶), skeletal muscle relaxing (Parry et al., 1993 ▶), anti-inflammatory (Agyare et al., 2015 ▶; Askari et al., 2016 ▶; Chan et al., 2000 ▶; Kaveh et al., 2017 ▶) and pain-relieving effects (Hajzadeh et al., 2004 ▶), anti-hyperlipidemic (Zidan et al., 2014 ▶), antidiabetic (El-Sayed 2011 ▶), endothelial dysfunction improving (Lee et al., 2012b ▶), vascular inflammation improving (Lee et al., 2012a ▶) and anti-histamine and β- adrenergic stimulatory (Boskabady et al., 2016 ▶; Hashemzehi et al., 2016 ▶) activities. A lower incidence of cancer and heart diseases has also been reported in areas where this plant is consumed (Naeem and Khan, 2013 ▶). P. oleracea contains several active compounds including flavonoids, omega-3 fatty acids, polysaccharide, alkaloids, coumarins, cardiac glycosides, anthraquinone glycosides (El-Sayed 2011 ▶), vitamins A , C and E, β-carotene, melatonin, dopamine, noradrenalin, oxalates and minerals (Ca^2+^, K^+^, Zn^2+^, and Na^+^) (Lim and Quah 2007 ▶; Liu et al., 2000 ▶; Simopoulos et al., 2005 ▶). Several studies have reported that Po seeds have strong antioxidant effects due to the presence of antioxidant components such as glutathione, omega-3 fatty acids, ascorbic acid, alpha tocopherols, kaempferol, quercetin, apigenin, beta-carotene, oxalate and melatonin (Zhou et al., 2015 ▶) (Chen et al., 2012 ▶; Dkhil et al., 2011 ▶; Yang et al., 2009 ▶). 

Yuan-cui X reported that treatment of rats with *P. oleracea *reduced serum angiotensin II levels (Yuan-cui, 2011 ▶). Inhibition of angiotensin converting enzyme activity by methanolic extract of Po was also reported by Geun-Pyo et al., (Choi et al., 2002 ▶). 

As mentioned above, there is an association between hyperthyroidism, RAS and oxidative stress and treatment with antioxidant agents and angiotensin receptor blockers can reduce cardiovascular effects of hyperthyroidism. Therefore, the aim of this study was to compare the effect of *P. oleracea* seeds extract as a herbal antioxidant and valsartan as an angiotensin type 1 blocker, on cardiac function in rats with levothyroxine-induced hyperthyroidism by assessment of echocardiographic and hemodynamic parameters.

## Materials and Methods

**Animals**

Male Wistar rats weighing 220-250 g, were obtained from animal house of Mashhad University of Medical Sciences, Mashhad, Iran. The Animals were maintained in plastic cages (ﬁve rats / cage) and they had free access to a standard diet and tap drinking water. They were housed under standard laboratory conditions (constant temperature at 24 ± 2°C with 12/12hr light/dark cycles) during the experiment. Study protocol was approved by the Ethics Committee of Mashhad University of Medical Sciences.

**Plant materials**

*P. oleracea* (Po) seeds were purchased from a local herb store in Mashhad, Khorasan Razavi province, Iran and identified by School of Pharmacy, Mashhad University of Medical Sciences (Herbarium No. 240-1615-12). Po seeds were powdered and 100 grams of powder was dissolved in 70% ethanol and placed in a Soxhlet extractor. The leading extract was condensed under reduced pressure and kept at 4°C until use. The weight of dried extract was 6.5 g, so the yield of the extract was 6.5% (w/w). The extract was dissolved in distilled water to prepare a dose of 400 mg/kg of the extract.

**Experimental design**

Forty Wistar rats were randomly divided into four groups (n=10): control group, levothyroxine group (T_4_), T_4_ plus valsartan group (T_4_-Val), and T_4_ plus hydro-alcoholic extract of the *P. oleracea* seeds (T_4_-Po). Control group received normal saline (intraperitoneally (ip) daily. Levothyroxine was given (100µg/kg/day, ip) to other three groups for 4 weeks. Valsartan (8mg/kg/day) and the extract of Po seeds (400mg/kg/day) were administrated orally by gavage during the last 2 weeks of the treatment period. Animals were weighted every week to assess weight changes during the treatment period (4 weeks). All doses of drugs were determined based on previous studies (Basset et al., 2000 ▶; Kim et al., 2012 ▶; Lee et al., 2012b ▶; Su et al., 2008 ▶). At the end of the experiment (on the 28th day), the echocardiographic and hemodynamic parameters were assessed while animals were anesthetized. Then, the thorax of animals was opened and the heart was rapidly removed and weighted.

**Thyroid hormones measurement**

 After echocardiographic and hemodynamic measurements, blood samples were collected, through a PE 50 catheter which was inserted into the right carotid artery. The blood samples were centrifuged at 3000 rpm for 15 min; blood serum was segregated and maintained at -20°C until measurement of hormones levels. Serum levels of free T_4_, T_3_, and T_4_ were measured by radioimmunoassay kits (RIAK, Korea) according to the protocols of kit manufacturer. Intra assay coefficient of variation was < 2.5%.

**Echocardiography**

At the end of treatment period, animals were anesthetized by ketamine (90 mg/kg, ip) and xylazine (10 mg/kg, ip) and were placed in supine position; next, echocardiography gel was applied to left hemi thorax and transducer was placed on the left hemi thorax. M-mode echocardiograms were attended from short-axis views of the left ventricle (LV) at the level of papillary muscles to determine ejection fraction (LVEF), fractional shortening (FS), and internal diameters during systole (LVESD) and diastole (LVEDD) using a Fukuda Denshi Ultrasound UF model 4300R equipped with a 9-MHz electronic transducer. Mean of three successive cardiac cycles measurements were recorded.

**Measurements of hemodynamic and cardiac hypertrophy development**

 Measurement of cardiac hemodynamic parameters was performed before the animals were sacrificed. The rats were anesthetized using ketamine 90 mg/kg and xylazine 10 mg/kg, i.p, and the right carotid artery was exposed and cannulated with a PE 50 catheter which was connected via a pressure-transducer to an amplifier. The catheter was moved forward into the left ventricle (LV) to record the left ventricular systolic pressure (LVSP, mmHg) and the left ventricular end-diastolic pressure (LVEDP, mmHg). Data were recorded over 10 min and analyzed with lab chart software (Blood Pressure Module). 

**Statistical analysis**

The data were expressed as mean±SEM. Kolmogorov-Smirnov test was used for the assessment of normality and statistical analysis was done by using one-way ANOVA and repeated ANOVA and LSD’s *post hoc* test to detect differences among groups. A p<0.05 was considered statistically significant.

## Results

**Thyroid hormones concentration**

 Four-week administration of T_4_ significantly increased serum free T_4_ level in T_4_ group (p<0.001 compared to control group); however, increases in T_4 _–Val group were not significant. On the other hand, free T_4_ level significantly decreased in T_4_–Po group as compared to T_4_ group (p<0.01); free T_4_ level in T_4 _–Po group was not statistically different from that of control group (Table 1).

The level of T_4_ in T_4_ and T_4 _–Val groups were increased by 12% and 19%, respectively in comparison with control group. The level of T_4_ in T_4 _–Po group was not statistically different from that of control group (Table 1).

The level of serum T_3_ in T_4_ and T_4 _–Val groups were not statistically different from that of control; but, in T_4_-Po group these levels significantly decreased (p<0.05) as compared to both control and T_4_ groups (Table 1).

**Hemodynamic parameters**

Heart rate (HR) was significantly increased in T_4_ group by 27% in comparison to control group (p<0.05) but in other groups, this value was not significantly different from that of the control group (Table 2). 

Heart weight was significantly increased only in T_4_ group when compared to control group (p<0.05) but in other groups, it was not statistically different from that of control group. Heart weight was significantly decreased in T_4 _–Po group as compared to both control and T_4 _groups (p<0.05 for both cases). Heart/body weight ratio was significantly increased in T_4_ group (p<0.001), in T_4_-Val (p<0.001) and T_4_-Po (p<0.001) as compared to control group (Table 2).

Data from cardiac catheterization showed that LVSP was significantly increased in T_4_-treated group when compared to control rats (p<0.001); however LVSP was insignificantlly reduced in T_4_–Val group by 14%, and T_4_ –Po group by 15% in comparison with T_4_ group (Table 2). LVEDP was insignificantly increased by 25% in the T_4_ group, 83% in T_4 _–Val, and 141% in T_4 _– Po group as compared with the control group (Table 2). 

**Echocardiographic parameters**

Echocardiographic evaluations showed that EF and FS were not significantly different among groups. End diastolic diameter was increased in T_4 _and T_4_-Val groups as compared to control group (p<0.01 for both cases). End diastolic diameter was decreased in T_4_-Po group as compared to T_4 _and T_4_-Val groups (p<0.01 for both cases). End systolic diameter was increased in T_4 _and T_4_-Val groups in comparison to control group (p<0.01 for both cases). End systolic diameter was also decreased in T_4_-Po group in comparison to T_4 _and T_4_-Val groups (p<0.001 for both cases) (Table 3).

**Table 1.** Thyroid hormones levels after 4-week treatment in different groups of rats.

**Parameters / Groups**	**Control**	**T**_4_	**T**_4_ **-Val**	**T**_4_ **-Po**
**Free T**_4 _ **(ng/dL)**	0.76±0.08	1.83±0.20***	1.27±0.30	0.68±0.11++
**T**_4 _ **(µg/dL)**	6.40±0.61	7.18±0.30	7.62±0.52	6.84±0.50
**T**_3_ ** (ng/dL)**	126.71±0.9	126.67±1.99	124.14±2.86	117.30± 2.42* +

All data are presented as the mean±SEM.

* p<0.05 and

*** p<0.001 indicate significant differences compared to control.

+ p<0.05 and

++ p<0.01 indicate significant differences compared to T_4_-treated group. SEM: Standard error of the mean, Val: Valsartan, Po: *Portulaca oleracea* (n=6-10).

**Table 2:** Changes in heart weight, heart weight/ body weight and hemodynamic parameters in different groups of rats

**Parameters / Groups**	**Control**	**T**_4_	**T**_4_ **-Val**	**T**_4_ **-Po**
**Heart weight (mg)**	855.45±16.64	913.33±12.51*	876.66±20.01	825.00±22.91++
**Heart/body weight (mg/g)**	2.97±0.04	3.37±0.06***	3.29±0.06***	3.29±0.06***
**HR (bpm)**	189.03±6.72	240.27±16.10*	211.27±12.89	203.10±11.87
**LVSP (mmHg)**	99.72±4.40	130.11±3.22***	111.73±3.76	110.64±4.18
**LVEDP (mmHg)**	1.29±1.54	1.52±2.10	2.44±1.65	2.91±1.77

All data are presented as the mean±SEM.

* p<0.05 and

*** p<0.001 indicate significant differences compared to control.

++ p<0.01 indicates significant differences compared to T_4_-treated group. SEM: Standard error of mean; Val: Valsartan; Po: *Portulaca oleracea*; LVSP: left ventricular systolic pressure; LVEDP: left ventricular end-diastolic pressure; and HR: heart rate (n=6-10).

**Table 3:** The results of echocardiography in different groups.

**Parameters/ Groups**	**Control**	**T**_4_	**T**_4_ **-Val**	**T**_4_ **-Po**
**EF,%**	66.18±1.63	62.70±1.86	60.67±1.85	66.25 ±3.02
**FS,%**	31.27±1.56	28.30±1.61	27.33±1.78	31.75±2.73
**EDD, cm**	0.65±0.03	0.80±0.03**	0.80±0.71**	0.57±0.31++ ××
**ESD, cm**	0.43±0.02	0.56‌±0.02**	0.61±0.06**	0.37±0.041+++ ×××

All data are presented as mean±SEM.

** p<0.01 indicates significant differences compared to control;

+ p<0.05,

++ p<0.01, and

+++ p<0.001 indicate significant differences compared to T_4-_treated group.

×× p<0.01 and

××× p<0.001 indicate significant differences compared to T_4_- Val; SEM: Standard error of mean; Val: Valsartan, Po: *Portulaca oleracea*; EF: Ejection fraction; FS: Fractional shortening; EDD: End diastolic diameter; and ESD: End systolic diameter (n=6-10).

**Body weight**

**Figure 1:**
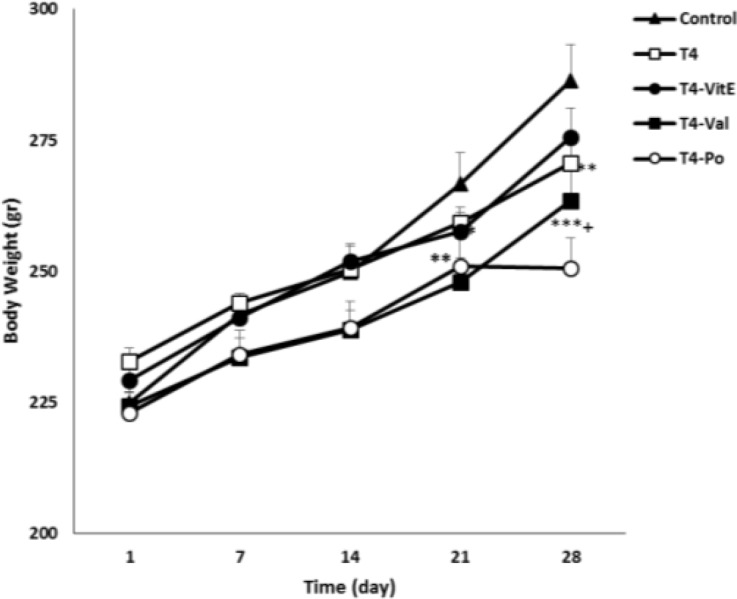
Comparison of the body weight during the 4 weeks among different groups using repeated measure ANOVA. All data are presented as mean±SEM. *p<0.05, **p<0.01 and ***p<0.001 indicate significant differences compared to control group. +p<0.05 indicates significant differences compared to T_4 _group. SEM: Standard error of mean; Val: Valsartan; and Po: *Portulaca oleracea* (n=10)

Body weight changes during the 4 weeks of experiment are presented in Figure 1. Baseline body weights were not different among groups. After 3 weeks, the body weights in T_4_-Val and T_4_-Po groups were significantly lower than those of control group (p< 0.01 and p<0.05, respectively). After 4 weeks, the body weights were significantly decreased in T_4_-Val and T_4_-Po groups as compared to control group (p<0.01 and p<0.001, respectively). It was also significantly decreased in T_4_-Po group in comparison to T_4_ group (p<0.05).

**Figure 2:**
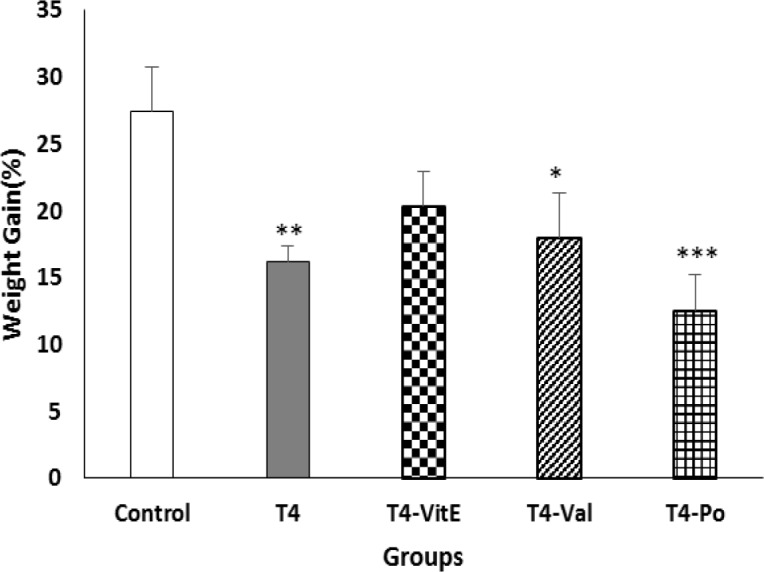
Comparison of the body weight gain percentage in different groups. All data are presented as mean±SEM. *p<0.05, **p<0.01 and ***p<0.001 indicate significant differences compared to control group. SEM: Standard error of mean, Val: Valsartan; and Po: *Portulaca oleracea *(n=10)*.*

The percentage of weight gain was decreased in T_4_, T_4_-Val and T_4_-Po groups in comparison to control group (p<0.01, p<0.05 and p<0.001 respectively; Figure 2).

## Discussion

The key findings of this study are: I) four-week treatment with levothyroxine produces signs and symptoms of hyperthyroidism including cardiac hypertrophy, increased HR and LVSP, but decreased weight gain; II) treatment with the hydro-alcoholic extract of Po seeds and valsartan reduced LVSP and HR but did not improve cardiac hypertrophy; III) treatment with Po seeds extract improved cardiac function by lowering HR, LVSP, and improving EF and FS.

Cardiovascular system is directly and indirectly affected by thyroid hormones. The direct effects of thyroid hormones on cardiovascular system are exerted at the cellular level through genomic and non-genomic routs. Thyroid hormones regulate transcription of cardiac–specific proteins involved in cardiac contraction and cardiac relaxation (Vargas-Uricoechea and Sierra-Torres 2014 ▶). Non-genomic effects of thyroid hormones include activation of intracellular kinase pathways that can alter myocardial contractility and relaxation, cardiac output, and blood pressure (Fazio et al., 2004 ▶). Thyroid hormones indirectly affect cardiovascular system by increasing thermogenesis but decreasing vascular resistance and cardiac afterload that lead to increases in cardiac output and positive inotropy (Vargas-Uricoechea and Sierra-Torres 2014 ▶). Thyroid hormones also affect cardiovascular system by activation of sympathetic system and renin-angiotensin systems. Thyroid hormones also stimulate erythropoietin secretion. High levels of thyroid hormones activate these pathways that results in increased blood volume and oxygen consumption leading to increased heart rate and cardiac output. These metabolic and hemodynamic changes can cause cardiac hypertrophy (Martinez 2016 ▶).

Previous studies reported that renin-angiotensin systems have an important role in hyperthyroidism-induced cardiac hypertrophy. It has been reported that treatment with T_4_, activates renin-angiotensin system and increases levels of renin and angiotensin II in the heart (Kobori et al., 1997 ▶). It was reported that treatment with thyroxin causes cardiac hypertrophy in rats and the drug cilazapril, an angiotensin converting enzyme inhibitor, prevented cardiac hypertrophy (Asahi et al., 2001 ▶). It was also reported that treatment with L-thyroxin induced cardiac remodeling and irbesartan prevented cardiac remodeling (Kim et al., 2012 ▶). So, angiotensin receptor blockers can be used for treatment of cardiac dysfunction caused by hyperthyroidism. Araujo et al., reported that L-thyroxin administration for 4 weeks caused cardiac hypertrophy, increased LVSP and LVEDP, increased lipid peroxidation and carbonyls, and decreased glutathione (Araujo et al., 2006 ▶). This study showed that hyperthyroidism can induce cardiac dysfunction and oxidative stress. Araujo et al., also showed that oxidative stress has an important role in hyperthyroidism-induced cardiac hypertrophy, and antioxidant agents reduced reactive oxygen species (ROS) and nitric oxide synthase (NOS) isoforms, nitric oxide metabolites, AT1/AT2 and cardiac hypertrophy (Araujo et al., 2008 ▶). These findings demonstrated that antioxidant agents may be effective in treatment of cardiac dysfunction induced by hyperthyroidism, by reducing the activity of cardiac renin-angiotensin system and reducing oxidative stress in the heart. 

In our study, four-week treatment with T_4_ significantly increased serum free T_4_ level, while administration of valsartan reduced the serum free T_4 _level but it was not significant compared to T_4_ group. *P. oleracea* seeds extract prevented the increases in free T_4_ and decreased T_4_ and T_3_ levels. Ashtiani et al., reported that *P. oleracea* increased T_3_ and T_4_ levels in rats treated with high-fat diet which is in contrast with our finding.

In the present study, levothyroxine treatments decreased body weight gain percentage in all groups treated with levothyroxine. The body weight loss is a primary sign of hyperthyroidism and it may be due to increased peripheral metabolism (Danzi and Klein, 2012 ▶). It has been demonstrated that treatments with thyroid hormones increase the basal metabolic rate and oxygen consumption in tissues (Danzi and Klein 2012 ▶; De Luise and Harker 1989 ▶). The results of our study also showed that the group treated with *P. oleracea* seed extract has the minimum weight gain. A previous study demonstrated that Po seeds reduces body weight and BMI in diabetic patients (El-Sayed, 2011 ▶). In many countries, Po is commonly used for weight loss. Po contains vitamin B1 or thiamine, and niacin, which are necessary as coenzymes for converting proteins, fat, and carbohydrates into ATP. Furthermore, Po contains noradrenalin which has lipolytic effects and could be effective in losing weight. In addition, the effect of Po seeds on body weight may be due to its effect on reducing insulin receptor sensitivity (El-Sayed, 2011 ▶). Thus, these properties of Po may affect the body weight. 

In the present study, a significant increase in heart weight/body weight ratio in all groups treated with levothyroxine was evident. Several studies have shown that treatment with levothyroxine induces cardiac hypertrophy (Araujo et al., 2008 ▶; Basset et al., 2001 ▶; Sabri et al., 1998 ▶). Heart weight was significantly increased in T_4_ group but treatment with valsartan and Po prevented increases in heart weight. In addition, our results showed that HR and LVSP increased in T_4_ group; treatment with valsartan and Po significantly prevented HR and LVSP elevation. Levothyroxine decreased EF and FS but increased EDD and ESD. Treatment with *P. oleracea* improved EF and FS and decreased EDD and ESD.

Several studies have shown that angiotensin receptor type I blockers are more effective than β-blockers in improving cardiac hypertrophy induced by hyperthyroidism (Asahi et al., 2001 ▶; Kim et al., 2012 ▶); but, in our study, two-week treatment with valsartan could not significantly decrease cardiac hypertrophy. This may be due to short period of treatment; however, valsartan lowered HR and LVSP. Several studies have shown that hyperthyroidism induces production of reactive oxygen species and impairs antioxidant systems (Bednarek et al., 2005 ▶; Guerra et al., 2005 ▶; Moreno et al., 2005 ▶). Araujo et al., showed that there is a positive correlation between cardiac hypertrophy and oxidative stress in hyperthyroidism (Araujo et al., 2006 ▶) and vitamin E decreases the expression of proteins which are involved in cardiac hypertrophy (Araujo et al., 2008 ▶). Our results showed that Po seeds extract prevented the increases in heart weight, heart rate, and LVSP. Also it improved cardiac function which can be attributed to its antioxidant properties. Lee et al., reported that in db/db mice, treatment with Po lowered systolic blood pressure which is in agreement with our results (Lee et al., 2012b ▶). *P. oleracea* contains melatonin which has free-radical scavenging properties and also contains flavonoids, phenolic compounds and ω-3 fatty acids with strong antioxidant effects (Liu et al., 2000 ▶; Simopoulos et al., 2005 ▶; Yang et al., 2009 ▶). The effects of Po seed extract in our study on cardiac function can be attributed to these antioxidant compounds.

In conclusion, our results showed that Po seeds extract and valsartan were effective in lowering of heart weight, HR, and LVSP in rats treated with levothyroxine; however, Po seed extract was more effective and it also improved EF and FS in comparison with valsartan. Therefore, usage of Po in crude form or in formulations could be suggested to be used for treatment of hyper function state of heart due to hyperthyroidism.
